# Current and future perspectives of an early diagnosis of cognitive impairment

**DOI:** 10.3389/fneur.2023.1171681

**Published:** 2023-04-05

**Authors:** Maurizio Giorelli

**Affiliations:** Operative Unit of Neurology, ASL BT, Barletta, Italy

**Keywords:** dementia, Alzheimer, rapid progressive dementia, diagnosis, therapeutics

## Introduction

Cognitive decline is a significant problem which will continue to exacerbate in the coming years. Estimates by the World Health Organization predict progressive aging of the global population in the coming decades, resulting in an increase in the prevalence of dementia in both industrialized and non-industrialized nations. This will cause significant strain on society, as healthcare systems will have to provide an increasing number of dementia patients with costly continuous assistance and the most suitable treatments for their fragile condition (1).

Until recently, the onset of cognitive impairment was considered inevitable due to the lack of availability of truly effective therapy. Family doctors and other healthcare workers could provide little assistance despite the legitimate need for healthcare and support for patients and their relatives. This unavoidability was linked to the identification of cognitive impairment with Alzheimer's disease (AD), a neurodegenerative disease for which only drugs and supplements with a symptomatic effect could be prescribed so far. Doctors commonly ignored the request for diagnostic investigations by patients, even in cases of mild cognitive impairment (MCI) or subjective cognitive impairment, postponing examinations based on the postulated incurability of the disease (2).

## Usefulness of an early differential diagnosis of cognitive impairment

The etiology of early onset cognitive impairment cannot be assumed, regardless of the patient's age. Although the prevalence of AD correlates with age, cognitive impairment may also be caused by an easily reversible iatrogenic, dysmetabolic, or structural condition (Table 1) (3). Among patients with Rapid Progressive Dementias (RPDs), potentially treatable conditions such as encephalitis or toxic-metabolic encephalopathies accounted for a whole of almost 30% (4). Delirium is an acute cognitive disturbance that causes a severe and fluctuating deficit in attention or thoughts. This condition can be provoked by mild fever, dehydration, or drug intoxication in sensitive patients, such as those affected by chronic vascular encephalopathy. Electrolyte disturbances such as hypo or hypernatremia and calcemia are also very common in the elderly and represent completely reversible causes of cognitive impairment (4). The most frequent cause of cognitive dysfunction is iatrogenic, including the intake of benzodiazepines (BDZ) or drugs with primary or secondary anticholinergic effects. Hypovitaminosis of B12 or of other vitamins in the B complex is frequent in older patients, in the weak, or in patients suffering from primary or iatrogenic feeding or absorption disorders. Additionally, thiamine (vit B1) deficiency can cause an amnesic-confabulatory syndrome associated with ophthalmoparesis (Wernicke' s syndrome), which is easily resolved with thiamine supplementation. Hormonal disorders, such as thyroid hormone deficiency, hypoadrenalism, and internal medicine conditions, such as hyperammonaemia caused by liver failure and anemia, are also easily diagnosed, and treated. Central Nervous System infections may also have an acute-subacute course and can be easily diagnosed and treated. Bacteria and other infectious organisms can reach the brain and meninges in several ways: hematogenous spread, penetrating head wounds, direct extension of cranial infections (e.g., sinusitis, osteomyelitis). Infective agents include viruses, bacteria, micetes, parasites such as Herpes Viruses, West Nile, Toxoplasma Gondii, Mycobacterium Tubercolosis, Treponema Pallidum, HIV-encephalopathy or Tropheryma Whippelii. Furthermore, the incidence of autoimmune encephalitis is increasing, triggered by sporadic para-infectious, or para-vaccine synthesis of an antibody that recognizes proteins usually with channel function (such as anti-N-methyl-D-aspartate, anti- alpha-amino-3-hydroxy-5-methyl-4-isoxazolepropionic acid, or anti-leucine-rich, glioma inactivated 1) on the neuronal surface. Production of these antibodies induces a severe neurological syndrome, in which subacute cognitive impairment is only a part of the symptomatologic picture. Although serious and potentially fatal, autoimmune encephalitis can be effectively treated with immunomodulatory drugs, such as corticosteroids, plasmapheresis, and vein IgG, often leading to complete remission. In the differential diagnosis of recent onset cognitive impairment, mood and thought disturbances as well as focal epileptic syndromes frequently arise from the temporal lobe. Ensuring a timely diagnosis allows physicians to prescribe a specific therapy, which may be decisive for the outcome. Structural causes of cognitive impairment include subdural haematomas, normotensive hydrocephalus, and space-occupying lesions, such as benign and malignant tumors. Some of the latter conditions may be effectively treated by the positioning of a ventricular shunt, evacuation, or removal treatments (4).

**Table 1:** Dementing pathologies classified by main symptoms, clues to suspicion, ideal time to diagnosis and main exams needed.

**Main symptoms (other than cognitive** ^*^ **)**	**Onset**	**Possible diagnosis**	**Ideal time to diagnosis**	**Diagnostic clues**	**Diagnostics**	**Possible outcome**
**Slow progressive amnestic (sometimes non amnestic) impairment**	**Months, sometimes weeks**	**Alzheimer**	**In the pre-symptomatic phase**	**Screening**	**Brain CT, MRI, Aβ-PET, CSF, blood exams, AI, COT**	**Treatable**
Fluctuating deficit of attention	Hours	Delirium	At onset	Dehydration, fever, dyspnoea, oedemas, pain, constipation, drugs intake	History collection, careful examination, blood and radiologic exams	Reversible
Amnestic condition, confabulation, diplopia	Days	Wernicke's encephalopathy	At onset	History of alcoholism, voluntary or obliged fasting	Blood exams, MRI	Reversible
Lack of initiative, behavioral changes, dishinibition	Days, weeks	Neurosyphilis, vascular dementia	At onset	Vascular risk factors, history of risky sex behaviors	CSF VDRL, TPHA, brain CT or MRI	Treatable
Mood instability, insomnia, self-devaluation	Weeks	Psychiatric disorders	At onset	Onset of psychiatric symptoms	Careful examination and history collection	Treatable
Apathy, weight loss or gain, tremor, variation in hair distribution	Weeks	Hypo/hyper cortisolism or thyroidism	Before somatic changes	Somatic changes	Blood exams, dosage of FT3, FT4, TSH, ACTH, cortisol	Reversible
Epilepsy, movement disorders	Days, weeks	Autoimmune or infectious encephalitis	At onset	Sudden onset of epileptic episodes	Brain MRI, EEG, paraneoplastic and channel antibodies	Treatable/reversible
Headache, and focal deficits	Weeks, months	Subdural haematoma, cerebral occupying lesions	New headache	Headache onset	Brain CT and MRI	Treatable/reversible
Sphinteric disfunction, gait disorders	Weeks, months	Normotensive hydrocephalus	Gait disorders	Whenever cognitive impairment starts	Brain CT, MRI, tap test, invasive CSF monitoring	Treatable/reversible
Paroxysmal episodes of confusion or staring	Days, weeks	Temporal epilepsy	When paroxysms start	Paroxysms of awareness suspension	Brain MRI, EEG, paraneoplastic and channel antibodies	Treatable
Generalized asthenia, confusion, irritability, hallucinations	Days, weeks	Drug intoxication or withdrawal	At onset	History of drug or substance abuse	Slow drug or substance withdrawal	Reversible
Bradykinisia	Weeks, months	Parkinson's disease	At onset	Slowness of movements and rigidity	Brain CT, MRI, dopamine transporter imaging	Treatable

* Cognitive impairment may be either amnesic or non-amnesic, involving language, attention, visuo-spatial skills, executive functions, orientation, or praxis. Treatable, treatment available but residual deficits are possible, Reversible: complete return to baseline because of timely and specific treatment. Acronyms not previously described: AI, Artificial Intelligence; COT, Computerized Optical Tomography; CSF, Cerebro SpinalFluid.

## Perspectives of an early diagnosis of AD-related cognitive impairment

Of all dementia cases, AD accounts for about 62%, Lewy body disease for 15%, vascular dementia for 6%, mixed dementia (AD+ vascular) for 6%, and other conditions for 11%. Cognitive impairment may also be related to rare neurodegenerative diseases such as Frontotemporal Dementia (FTD), Multiple System Atrophy (MSA), Progressive Supranuclear Palsy, Corticobasal Degeneration (CBD) which sometimes may mimic clinic presentation of AD. As the most frequent form of dementia is AD, it has warranted significant research efforts to identify cost-effective therapies. Neuropathological studies using *in vitro* and *in vivo* disease models have identified the pathogenic cascade in AD that leads to the formation of a pathological protein, Aβ-amyloid (Aβ), which accumulates to form “plaques”, probably leading to neuronal death. Another landmark of AD is the formation of neurofibrillary tangles formed by hyperphosphorylated tau protein. Many other proteins are involved in this pathogenic process, including amyloid precursor protein, presenilin 1 and 2, apolipoprotein E (APOE)4, and tau protein. This system is also supported by the mechanisms of inflammation and the formation of oxygen free radicals (1). Most therapeutic approaches implemented to date concerning disease-modifying drugs (DMDs) have targeted the molecules responsible for the pathogenic enzymatic cascade, with most attempting to inhibit their activity and promote neuronal protection. The targets include amyloid-beta, tau, APOE4, lipoprotein receptors, metabolism, vessels, growth factors, hormones, synaptic plasticity, and neuroprotective pathways.

Although AD has long been considered an incurable disease characterized by a poor course and prognosis, the advent of DMDs for AD has changed this view. Two molecules have shown efficacy in recently completed clinical trials. Aducanumab and Lecanemab are both humanized monoclonal antibodies, the first directed against plaques and Aβ oligomers and the second against Aβ protofibrils (Table 2) (5). They both remove Aβ proteins associated to brain plaques and slow the rate of cognitive impairment but do not reverse it at all. Either Aducanumab and Lecanemab have been approved by FDA for treatment of AD in USA but not in the European Community. Moreover, the onset of neuropathological changes within the brain and the onset of symptoms are separated by several years. Maximum benefit would be achieved if the DMDs therapy were initiated in this prodromal, asymptomatic phase, or at initial symptom onset. The guidelines issued by the Food and Drug Administration in 2013 suggested enrolment of AD patients who were in the initial stages of the disease in clinical trials (1), although some ongoing trials on anti-amyloid antibody treatments included asymptomatic patients having Aβ deposition as assessed by brain Amyloid PET scan. Furthermore, the identification of such patients is an obstacle.

**Table 2:** List of drugs approved for treatment of AD.

**Name of drug**	**Mechanism of action**	**Effects**	**Approval**
Rivastigmine	Acetylcholinesterase inhibitor	Improves cognition and controls some behavioral symptoms	FDA, EU
Galantamine	Acetylcholinesterase inhibitor	Improves cognition and controls some behavioral symptoms	FDA, EU
Donepezil	Acetylcholinesterase inhibitor	Improves cognition and controls some behavioral symptoms	FDA, EU
Memantine	NMDA receptor antagonist	Delays progression of symptoms and allows patient to maintain daily functions	FDA, EU
Aducanumab	Monoclonal antibody against Aβ	Delays Aβ deposition and AD progression	FDA
Lecanemab	Monoclonal antibody against Aβ protofibrils	Delays Aβ deposition and AD progression	FDA

## Difficulties of early differential diagnosis

The diagnosis of cognitive impairment may include clinical evaluations, complex neuropsychological tests, blood chemistry tests, cerebrospinal fluid analysis, instrumental diagnostic tests such as Cranial Tomography (CT), brain Magnetic Resonance Imaging (MRI), brain Positron Emitting Tomography (PET) scans for specific markers, and genomic analysis (1). Given the large number of conditions that may trigger cognitive impairment, it is difficult to produce a diagnostic algorithm that encompasses all the diagnostic possibilities. Rather, the etiological diagnosis of cognitive impairment must take into consideration important “red flags”, such as the age of onset, the speed of the disease development, familiarity as well as the physiological, pathological, and pharmacological patient's history which must then guide the clinician toward the necessary tests.

## Challenges of early diagnosis in AD: Limits of biomarkers

Given the importance of early diagnosis of AD for both clinical trials and therapeutic practice, recruiting patients already in the clinical phase even if mild, (e.g., Mild Cognitive Impairment), could be suboptimal. Pathological processes which started years earlier in the brain could limit the magnitude of the therapeutic strategy or its effectiveness over time. Therefore, diagnosis should optimally be performed in the “pre-symptomatic AD” or “at risk of AD” phase to establish the most effective interventions even before symptoms appear (1, 5). In this context, enormous efforts have been devoted to searching for imaging or molecular biomarkers (6, 7) that could become both enrolment and outcome requirements in clinical trials aimed at verifying the efficacy of DMDs. Therapy for anemia, for example, may be established in a specific manner through blood sampling and specific tests, even in the absence of overt symptoms. Thus, screening tests are of high value. Ideal biomarkers in screening tests for Preventative Medicine must be sensitive, specific, accessible to all, low-cost, and non-invasive. Thus, an ideal biological marker would be derived from plasma. Although studies on dementing-neurodegenerative diseases have shown a considerable overlap of pathogenic proteins, supporting the idea that AD is a syndrome of many etiologies (8), a recent study confirmed validity and suitability of blood biomarkers to predict AD in a large cohort complying subjective cognitive impairment (9). In May 2022 FDA approved a test to easily detect Amyloid Beta 42/40 ratio (Aβ 42/40) and Apolipoprotein E proteotype (equivalent to ApoE genotype) in blood samples, Artificial intelligence approaches such as machine learning may facilitate the early diagnosis of AD using surrogate biomarkers through the recognition of voice profiles (10), beta-amyloid accumulation on brain PET (11), or certain characteristic profiles on brain MRI such as medial temporal atrophy (12). Although promising and non-invasive, machine learning approaches remain largely inaccessible and poorly understood. Recent research has further promoted the retina as a promising diagnostic target for AD. The brain and the retina share an embryonic origin, and researchers have identified the accumulation of Aβ in the retina in the early stages of AD. Furthermore, a correlation has been identified between the thickness of the retina with that of the parietal lobe cortex, suggesting that AD is not only a pathology of the brain, but also of the eye. AD patients exhibit a reduced optic nerve fiber layer thickness demonstrable on Computerized Optical Tomography (COT), a non-invasive and inexpensive method that can be easily used as a screening method for AD (6).

## Dementia heterogeneity, genes, proteins, the exposome, and the “threshold” effect

Dementia patients often exhibit neuropathological heterogeneity (8). The prevalence of AD increases with age, as the brain also begins to develop cerebrovascular disease and hippocampal sclerosis (2). AD is a multifactorial pathology which involves the competition of environmental risk factors with a multiplicity of genes. Modifiable risk factors include diabetes mellitus, hypertension, smoking, obesity, and hypercholesterolemia, while protective factors include a high level of education, Mediterranean diet, and physical exercise (13).

The interaction of numerous genes involved in neuronal plasticity in the determinism or protection from dementia is also very complex (14). The paradigm of molecular biology is that the function of proteins is determined by genes encoding for them. Mutated genes give rise to modified proteins, possibly having anomalous functions. Certain functions can be lost (“loss-of-function”) or acquired (“gain of function”). Extensive effort has been made to discover the interactions between pathological proteins, but the biological effect of a mutated protein is poorly predictable based on a deterministic, reductionist approach. The interactions of an altered protein are in fact determined by its “size” effect and by the differential expression of the proteins of its own metabolic network, which is also a proper factor of that protein (15). Proteins may also show a variable number of interactions within their network, as demonstrated by experiments in *Saccharomyces cerevisiae* models (16). The interactions of normal and pathological proteins are therefore subject to the laws of complexity rather than those of mechanics. The AD-exposome is the complex of all environmental factors to which we are exposed all life long and are relevant to genetic underpinnings of cognitive aging and AD (17). The exposome acts on genes to determine the result of cellular and, even more so, mechanisms of pathology within organs. The result of the neurodegenerative mechanisms therefore depends on the “threshold” factor of its determinants, which induces the expression of the pathology and can also explain the multiformity of its variants, including AD variants (7). This great pathogenetic variety may therefore determine resistance or a lower response to brain-clearing therapies, even if patients have a significant amyloid load. Therefore, it is reasonable to assume that proteins other than Aβ could be useful therapeutic targets. Therapeutic approaches aimed at limiting the damage induced by arteriosclerosis, blood-brain barrier dysfunction, and alpha-synuclein, which can interact with amyloid and abnormal tau in individuals with “sporadic AD”, should be considered. This approach is futuristic in a precision medicine context considering a panel of biomarkers as part of an individual pathology “fingerprint”.

## Future applications

The notion that cognitive impairment is an inevitable and incurable condition must be abandoned. The advent of Alzheimer's modifying therapies (Table 2), such as monoclonal antibodies against Aβ, might soon change the clinical course of patients with this disease. At least three different key factors are involved in successful addressing AD therapeutics: (1) diagnosis in a pre-symptomatic phase; (2) dissecting mixed pathology and identifying the correct molecular targets; (3) availability of cheap, predictive, accurate, not invasive, and widely accessible biomarkers. The knowledge of the correct diagnosis in advance with respect to the clinical onset of symptoms will allow physicians and patients to further stress modification of inter-linked pathologies such as diabetes, hypertension, and major vascular events which are also risk factors for neurodegenerative diseases.

The onset of cognitive decline, even if only subjective, can also hide a treatable but potentially progressing condition to a fatal event. The diagnosis of cognitive impairment requires accurate collection of clinical history, considerable attention, and a procedural algorithm, the complexity of which increases with the relevance of the symptomatology pleomorphism.

## Author contributions

The author confirms being the sole contributor of this work and has approved it for publication.
